# Comparison of the learning curves of the osteotomy guide robot and guide plate-based robot-assisted total knee arthroplasty

**DOI:** 10.1186/s42836-026-00388-5

**Published:** 2026-05-06

**Authors:** Xin Zhi, Te Liu, Peng Ren, Qingyuan Zheng, Ming Ni, Guoqiang Zhang

**Affiliations:** 1 Senior Department of Orthopaedics, Chinese PLA General Hospital, Beijing, 100142 China; 2 Department of Orthopaedics, Union Medical College Hospital, Chinese Academy of Medical Sciences & Peking Union Medical College, Beijing, 100730 China; 3 PLA Medical School, PLA General Hospital, Beijing, 100142 China

**Keywords:** Robot-assisted total knee arthroplasty, Guide plate-based robot, Osteotomy guide robot, Learning curve

## Abstract

**Background:**

This study compared the learning curves and clinical outcomes of osteotomy guide robot and guide plate-based robot-assisted total knee arthroplasty (TKA).

**Patients and methods:**

From January to May 2023, 100 patients were prospectively enrolled to receive either a guide plate-based robot or an osteotomy guide robot-assisted total knee arthroplasty. The thickness of the osteotomy planned by the robot and the actual thickness were recorded in real time during the operation, as was the time taken for each step in the operation, including bone registration and osteotomy. The SF-12, HSS score, and FJS of the patients before surgery and 6 weeks and 24 months after surgery were also collected.

**Results:**

For surgeon 1, the average operating time with the guide plate-based robot and osteotomy guide robot was 98.16 ± 9.68 and 118.52 ± 15.95 min, respectively; the difference was significant. The average time of the last 10 cases was shorter than that of the first 10 cases. The inflection points of the osteotomy learning curve of surgeon 1 with two robotic systems were at case 5 and case 9. The average operative times for Surgeon 2’s two robotic surgery groups were 104.52 ± 12.65 min and 105.76 ± 33.03 min, respectively. The inflection points of the osteotomy learning curves using the two robotic systems occurred at case 13, respectively. Patients who underwent guide plate-based robot or osteotomy guide robot-assisted TKA had similarly improved knee recovery, reflected in the SF-12, HSS score, and FJS.

**Conclusions:**

There was no significant difference in the osteotomy learning curve between the two robotic systems. The improvement in knee functional recovery was similar after the guide plate-based robot and the osteotomy guide robot-assisted TKA.

**Level of evidence:**

Level II.

## Introduction

The application of robotic-assisted total knee arthroplasty (TKA) has gained increasing prominence. Robotic-assisted TKA has been clinically demonstrated to facilitate precise osteotomy, accurate prosthetic implantation, and restoration of lower limb alignment [1, 2]. Additionally, it enables balanced extension-flexion gaps tailored to individual bone morphology and collateral ligament tension, thereby minimizing abnormal stress distribution and polyethylene wear [3, 4].

According to a mechanism-based classification, robotic systems are categorized into image-guided and image-free platforms, as well as direct osteotomy and template-guided types [5–8]. In direct osteotomy systems, an oscillating saw is mounted directly onto the robotic arm, enabling autonomous precision bone resection. While potentially offering higher accuracy, they alter established surgical workflows. Template-guided systems position cutting blocks via the robotic arm for indirect osteotomy. Intra-operatively, the robot navigates the block to planned coordinates, after which the surgeon manually performs resection with a handheld saw. This approach primarily tests the robot’s intra-operative tracking capability, though accuracy remains questionable [9, 10]. Currently, there is a paucity of comparative studies analysing the merits and limitations of these two robotic paradigms.

There is a learning curve involved in using robots. As the number of robot-assisted hip and knee arthroplasty surgeries increases, surgeons become increasingly proficient, and there is improved registration and osteotomy accuracy [11, 12]. Its assisted osteotomy function is realised by the pendulum saw module. The accuracy of registration, osteotomy, and pre-operative prosthesis planning is all related to the surgeon’s learning curve. A study from the United Kingdom shows that the learning curve regarding the total operating time of surgeons using the MAKO robot had an inflection point at 7 cases [1]. The osteotomy function of the guide plate-based robotic system is realised by the osteotomy guide plate module, including ROSA and TINAVI [8]. Previous research has indicated that the initial learning curve for the ROSA Knee System can be achieved in 6–11 cases for operative time [13]. Currently, there are no data on the learning curve of the two robotic systems performed by the same surgeon.

Therefore, this study compared the learning curves of the two robotic systems in guide plate-based robot and osteotomy guide robot-assisted TKA in terms of the operating time, time used for each step during the operation, patient follow-up, osteotomy accuracy, and osteotomy learning curve.

## Material and methods

For guide plate-based robotic systems, we have selected the TINAVI robotic system. This system currently has the largest number of documented surgical cases among pin-guided robotic platforms in Chinese orthopaedic surgery. The TINAVI robot is designed by the TINAVI company in China. The MAKO Robotic System is currently the most widely utilized osteotomy guide robotic system in international orthopaedic surgery. The MAKO robot and associated technologies were purchased by Stryker in 2013. A prospective observational study of TINAVI robot-assisted TKA was conducted between January and May 2023, enrolling 50 consecutive patients. And 50 patients undergoing MAKO-assisted TKA were consecutively enrolled during the identical timeframe. Complete 24-month follow-up data were obtained for all cohort participants. The inclusion criteria were patients diagnosed with knee osteoarthritis requiring surgical treatment, and all surgeries were performed by two surgeons. Exclusion criteria were patients undergoing simultaneous bilateral TKA, patients with knee joint infection or undergoing revision surgery, and incomplete intra- and post-operative data. The research protocol was approved by the Ethics Committee of Chinese PLA General Hospital (S2022-744-02).

### Surgical strategy

For both systems, a pre-operative hip-knee-ankle joint CT scan was performed. A standardized surgical protocol was implemented in all cases, featuring pre-operative digital planning via robotic systems, intra-operative gap balancing, and dynamic intra-operative plan modification. The pre-operative planning for the TINAVI robot shares similarities with the MAKO system in its intra-operative guidance interface, with both systems providing gap measurement data. Specifically, all guide plate-based robotic systems utilized Attune PS implants (Depuy). An osteotomy guides the robotic system employed for Triathlon PS prostheses (Stryker). Post-operative mechanical alignment deviations were maintained within 3° of neutral. Tourniquet application was strictly limited to ≤ 60 min for every procedure. All surgeons involved in the procedures have extensive experience with manual Triathlon and Attune prosthesis implantation. Two senior surgeons have over 10 years of experience in joint replacement, each performing more than 200 total knee arthroplasty procedures annually. Although they had no prior experience with MAKO or TINAVI robotic-assisted total knee arthroplasty, they have performed various robotic-assisted total hip arthroplasty procedures and are very familiar with robotic bone registration technology.

### Intra-operative observation indicators

During the operation, the total operating time, time of each step, planned osteotomy thickness, actual measured thickness, and complications during the operation were recorded. The time of each operative step was recorded by a dedicated person. The time from the start of implementation to the end of the operation is defined as the operating time. During the procedure, the surgeon used a vernier caliper to measure the thickness of each osteotomy fragment with an accuracy of 0.1 mm. A designated assistant recorded both the measured thickness of the osteotomy fragments and the planned osteotomy thickness displayed by the robotic system, and calculated the difference between the two values.

### Post-operative follow-up

All patients were followed by two independent doctors, with final composite scores calculated as the arithmetic mean of their evaluations. The patients were seen the day before the surgery, 6 weeks, and 24 months after the surgery was completed. The surgery was evaluated using the Short Form-12 Health Survey (SF-12), Hospital for Special Surgery (HSS), and Forgotten Joint Score (FJS).

### Data summary and analysis

The statistical analysis was performed using SPSS 21.0 (SPSS Inc., USA). For measurement data, normality was first tested. If the data were normally distributed, the measure was expressed as the mean ± standard deviation. Paired *t*-tests were used to compare the total operating times and the initial and final individual operating steps. The test level α-value was set to bilateral 0.05.

A cumulative sum control chart (CUSUM) was created with Microsoft Excel 2019 (Microsoft, USA) to calculate averages, differences, and cumulative sums.

$${\mathrm{CUSUM}}_{\left(1\right)}\;=\;\mathrm{osteotomy}\;\mathrm{time}\;{\left(\mathrm{OT}\right)}_{\left(1\right)}\;\mathrm{of}\;\mathrm{the}\;\mathrm{first}\;\mathrm{case}\;-\;\mathrm{average}\;\mathrm{osteotomy}\;\mathrm{time}\;{\mathrm{OT}}_{\left(\mathrm{mean}\right)},\;{\mathrm{CUSUM}}_{\left(\mathrm n\right)}\;=\;{\mathrm{OT}}_{\left(\mathrm n\right)}\;-\;{\mathrm{OT}}_{\left(\mathrm{mean}\right)}\;+\;{\mathrm{CUSUM}}_{\left(n-1\right)}$$

The calculation is continued until the last case.

Taking the ordinal number of surgical cases as the abscissa and the CUSUM value of the osteotomy time as the ordinate, a learning curve was plotted and fitted. When *p* < 0.05, it is judged that the curve fitting is successful, and R^2^ is used to judge the goodness of fit. The fixed point of the CUSUM fitting curve is used as the minimum number of cumulative surgical cases required to cross the learning curve.

## Results

The guide plate-based robot group comprised 50 patients (15 males, 35 females; average age, 65.99 ± 6.50 (range: 53–81) years. The osteotomy guide robot group included 50 patients (18 males, 32 females; average age, 66.71 ± 6.48 (range: 54–80) years. The Triathlon PS (Stryker, USA) prosthesis was used (56% and 44% of surgeries on the left and right sides, respectively). No statistically significant differences were observed in baseline demographic and clinical characteristics between the two robotic-assisted groups for either Surgeon 1 or Surgeon 2 (Table 1). The surgeries were completed by two surgeons. All participating surgeons are senior orthopaedic specialists, each performing over 200 knee arthroplasties annually. No nerve or vascular injuries occurred during surgery. None of the patients required a blood transfusion. All patients exhibited no evidence of prosthetic loosening or peri-prosthetic joint infection (PJI) throughout the follow-up period. All patients were able to walk on the first post-operative day.

**Table 1:** Demographics and baseline statistics

Surgeon	Characteristics	Guide plate-based robot group	Osteotomy guide robot group	
**Surgeon 1**	Age (years)	66.09 ± 6.20	66.80 ± 5.44	
	Gender: male (%)	8/25	10/25	
	Side: right (%)	7/25		11/25
	BMI (kg/m^2^)	26.58 ± 4.04	27.15 ± 2.99	
**Surgeon 2**	Age (years)	65.81 ± 7.10	66.48 ± 8.34	
	Gender: male (%)	5/25	8/25	
	Side: right (%)	10/25	11/25	
	BMI (kg/m^2^)	27.36 ± 3.21	26.13 ± 3.79	

### Learning curves of osteotomy guide robot and guide plate-based robot-assisted TKA

**Fig. 1:**
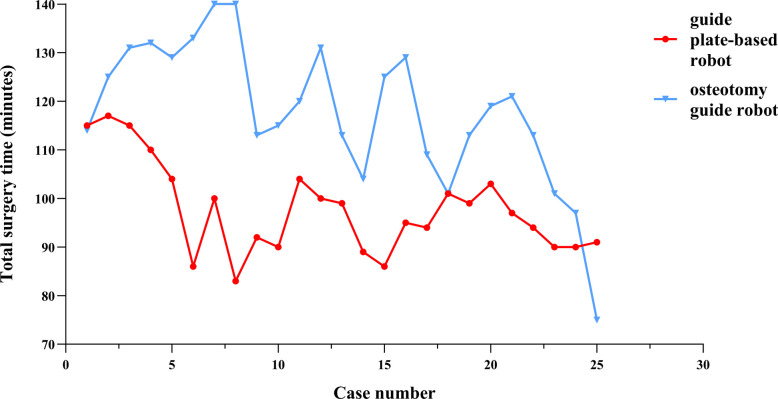
Flow chart of the total operation time of surgeon 1

**Fig. 2:**
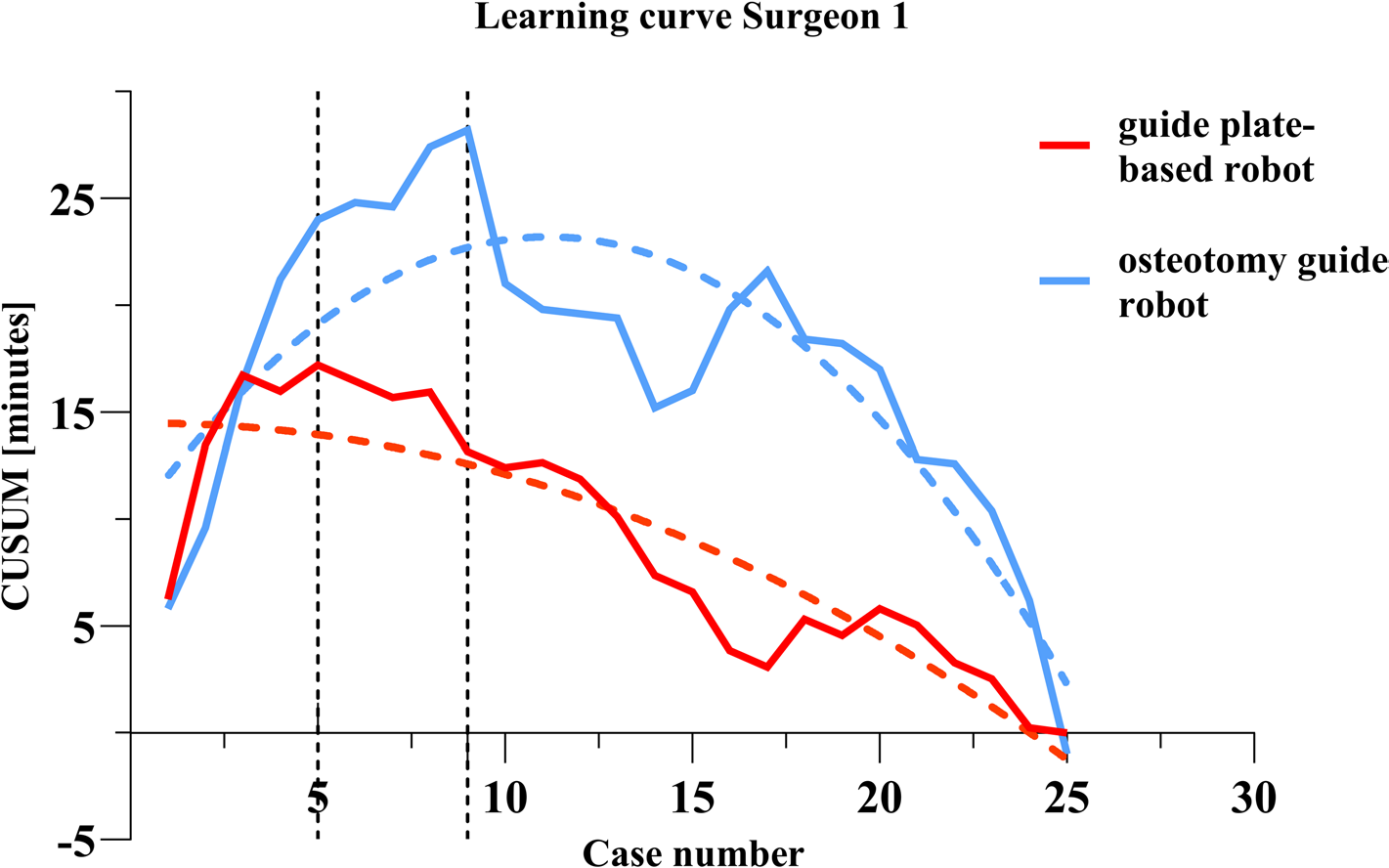
Learning curve of osteotomy time of surgeon 1

The total operating times of surgeon 1 using the guide plate-based robot and osteotomy guide robo*t* showed significant downward trends (Fig. 1). The average operating time with the guide plate-based robot and osteotomy guide robots was 98.16 ± 9.68 and 118.52 ± 15.95 min, respectively. The average operating time with the guide plate-based robot decreased from 101.2 ± 12.9 min in the first 10 cases to 95.4 ± 4.55 min in the last 10 cases (Table 2). The average operating time with the osteotomy guide robot decreased from 129.2 ± 11.94 min in the first 10 cases to 107.8 ± 15.24 min in the last 10 cases. In both the first and last 10 cases, the operating time was shorter in the guide plate-based robot group than in the osteotomy guide robot group, and the difference was significant. There was no significant change in the femoral or tibial registration times of surgeon 1 between the first and last 10 cases. In the guide plate-based robot group, the osteotomy time decreased from 9.00 ± 3.30 min in the first 10 cases to 7.10 ± 1.60 min in the last 10 cases, although there was no statistical difference. In the osteotomy guide robot group, the osteotomy time decreased significantly from 15.30 ± 3.97 to 11.50 ± 3.21 min. The osteotomy time for the first 10 cases differed significantly between the two robots, being significantly shorter in the guide plate-based robot group. The same was true for the osteotomy time of the last 10 cases. In the CUSUM analysis of osteotomy time, the fitting curves of the two groups were y = − 0.02618 × 2 + 0.03x + 14.46 (guide plate-based robot) and y = − 0.1088 × 2 + 2.42x + 9.69 (osteotomy guide robot). The osteotomy time of the guide plate-based robot group reached an inflection point at case 5 versus case 9 in the osteotomy guide robot group (Fig. 2).

**Table 2:** Operative data in patients undergoing two robotic-arm-assisted TKA

Surgeon	Operative stage (mins)	Guide plate-based robot-TKA		*p*-value	Osteotomy guide robot -TKA		*p*-value	*p*-value for comparison between two groups	
		**Cases 1–10**	**Cases 16–25**		**Cases 1–10**	**Cases 16–25**		**Cases 1–10**	**Cases 16–25**
**1**	Femur registration	1.85 ± 0.47	1.95 ± 0.44	0.642	4.20 ± 3.33	3.70 ± 3.34	0.781	0.052	0.146
	Tibia registration	1.95 ± 0.50	1.95 ± 0.37	1.000	3.80 ± 3.36	3.80 ± 3.36	1.000	0.122	0.141
	Osteotomy	9.00 ± 3.30	7.10 ± 1.60	0.124	15.30 ± 3.97	11.50 ± 3.21	0.003^a^	< 0.001^a^	0.008^a^
	Overall operating time	101.2 ± 12.9	95.4 ± 4.55	0.135	129.2 ± 11.94	107.8 ± 15.24	0.003^a^	0.001^a^	0.018^a^
**2**	Femur registration	2.10 ± 0.52	3.20 ± 1.38	0.040^a^	2.80 ± 1.23	2.60 ± 1.58	0.642	0.105	0.354
	Tibia registration	1.85 ± 0.34	2.10 ± 0.88	0.299	2.40 ± 1.07	2.80 ± 2.97	0.711	0.170	0.502
	Osteotomy	10.70 ± 2.50	9.10 ± 3.48	0.203	13.50 ± 3.41	9.60 ± 2.72	0.003^a^	0.062	0.722
	Overall operating time	104.00 ± 8.71	100.00 ± 14.26	0.443	114.10 ± 29.19	112.10 ± 18.72	0.829	0.342	0.146

^a^Indicates statistical significance

**Fig. 3:**
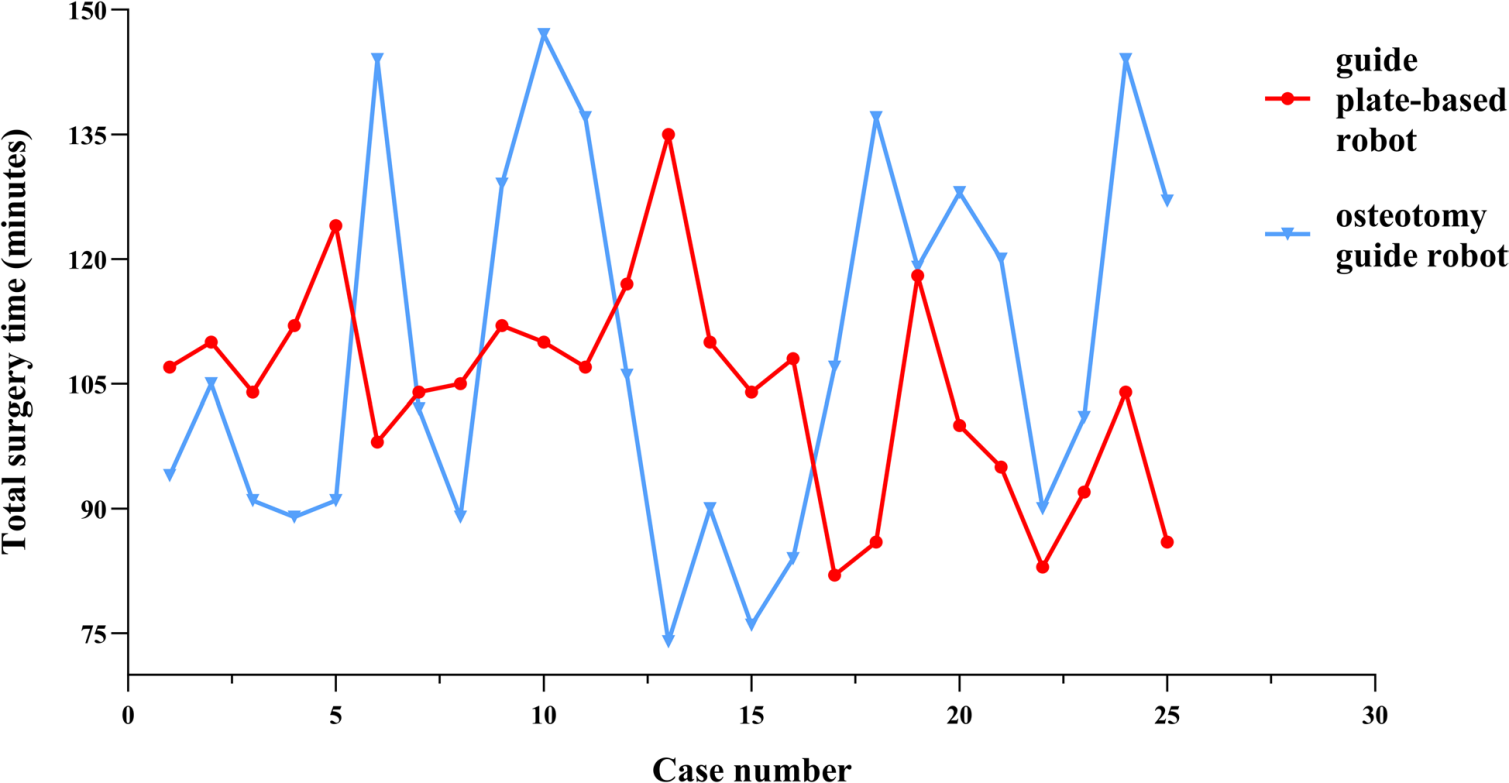
Flow chart of the total operation time of surgeon 2

**Fig. 4:**
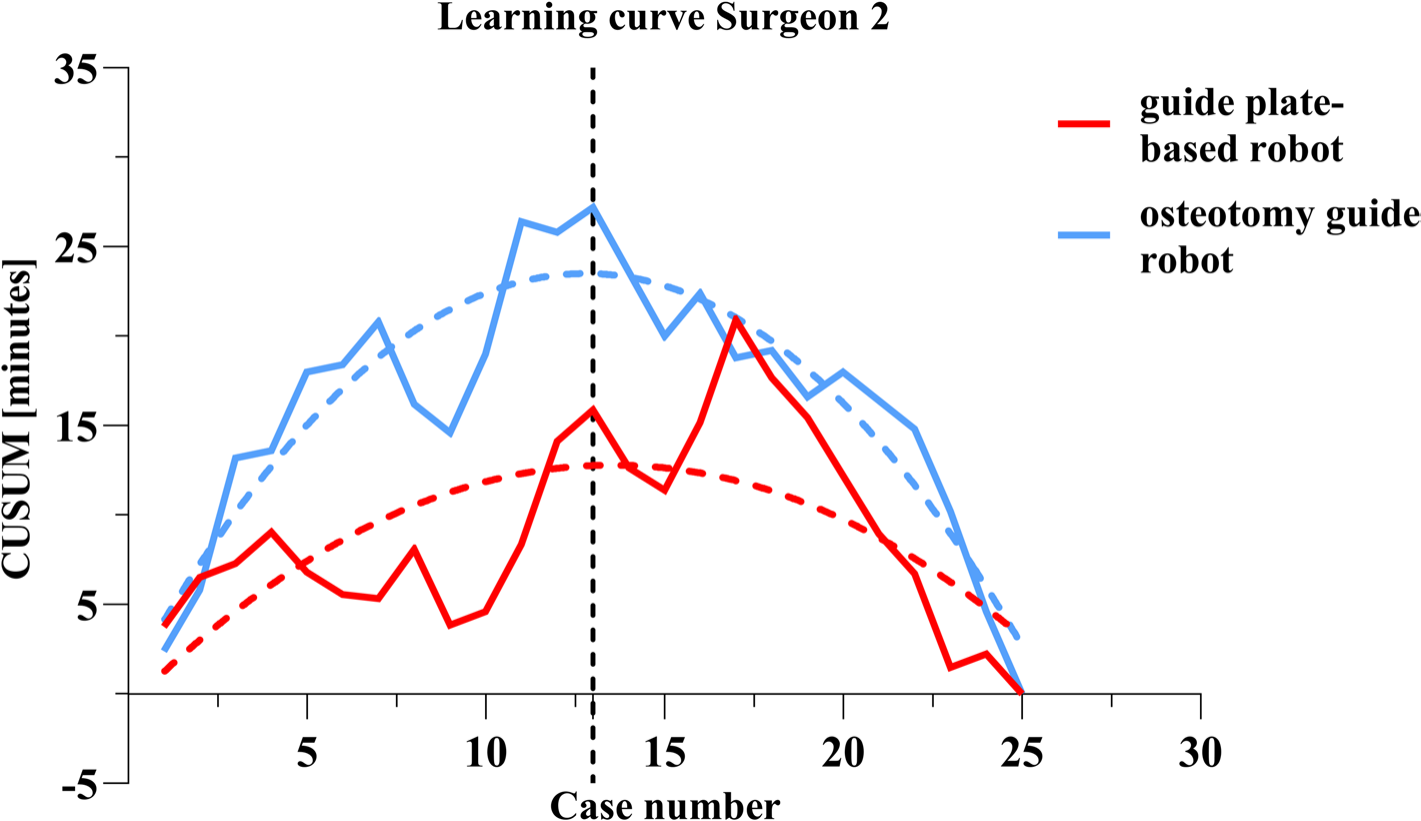
Learning curve of the osteotomy time of surgeon 2

For surgeon 2, the total operating times with both the guide plate-based robot and osteotomy guide robot robots fluctuated (Fig. 3). The average operating times with the guide plate-based robot and osteotomy guide robot robots were 104.52 ± 12.65 and 105.76 ± 33.03 min, respectively. The average operating time decreased from 104.00 ± 8.71 min in the first 10 cases to 100.00 ± 14.26 min in the last 10 cases in the guide plate-based robot group (Table 2), compared with a decrease from 114.10 ± 29.19 to 112.10 ± 18.72 min in the osteotomy guide robot group. There was no significant change in the femoral or tibial registration times of surgeon 2 between the first and last 10 cases. In the guide plate-based robot group, the osteotomy time decreased from 10.70 ± 2.50 min in the first 10 cases to 9.10 ± 3.48 min in the last 10 cases. In the osteotomy guide robot group, the osteotomy time decreased significantly from 13.50 ± 3.41 to 9.60 ± 2.72 min. The osteotomy time of the first 10 cases did not differ statistically between the two robots, although it was shorter with the guide plate-based robot; the same was true for the last 10 cases. In the CUSUM analysis of osteotomy time, the fitting curves of the two groups were y = − 0.0733 × 2 + 1.99x − 0.67 (guide plate-based robot) and y = − 0.1395 × 2 + 3.57x + 0.67 (osteotomy guide robot). The osteotomy time of the two robotic systems reached an inflection point at case 13 (Fig. 4).

The overall osteotomy accuracy with the guide plate-based robot was 0.39 ± 1.68 mm. Comparing the osteotomy accuracy of the first and last 10 cases, it improved from 0.61 ± 2.00 to 0.10 ± 1.58 mm, although there was no statistical difference. The errors in the osteotomy amount on the lateral side of the distal femur and medial and lateral sides of the posterior femur condyle were all < 0.1 mm, while the error in the osteotomy on the lateral side of the proximal tibia was greater, at 1.70 ± 1.58 mm (Table 3).

**Table 3:** Pre-operative planning of osteotomy accuracy in patients undergoing guide plate-based robot-assisted TKA

Osteotomy Position	Overall	Initial 10 cases	Last 10 cases	*p*-value
Lateral distal femur	0.01 ± 11.2	− 0.83 ± 1.45	0.01 ± 1.10	0.185
Medial distal femur	0.11 ± 1.87	0.41 ± 1.84	− 0.38 ± 1.35	0.443
Lateral posterior femoral condyle	0.05 ± 1.29	0.16 ± 1.53	− 0.66 ± 1.08	0.114
Medial posterior femoral condyle	0.08 ± 1.57	− 0.01 ± 1.95	0.25 ± 1.79	0.799
Lateral proximal tibia	1.70 ± 1.58	2.57 ± 1.43	1.68 ± 1.75	0.185
Medial proximal tibia	0.4 ± 1.89	1.38 ± 2.13	− 0.29 ± 1.40	0.153
Overall	0.39 ± 1.68	0.61 ± 2.00	0.10 ± 1.58	0.129

### Patient satisfaction with the osteotomy guide robot and guide plate-based robot-assisted TKA

In the guide plate-based robot group, the pre-operative patient HSS and SF-12 scores were 42.88 ± 2.91 and 44.22 ± 4.27, respectively, versus 43.46 ± 3.43 and 43.12 ± 3.89 in the osteotomy guide robot group. There was no statistical difference between the two groups. At 6 weeks post-operatively, the HSS and SF-12 scores were 65.1 ± 4.54 and 62.08 ± 4.80, respectively, in the guide plate-based robot group versus 63.2 ± 4.82 and 62.94 ± 4.82 in the osteotomy guide robot group. At 24 months post-operatively, the HSS and SF-12 scores were 78.96 ± 5.35 and 80.54 ± 4.67, respectively, in the guide plate-based robot group versus 80.56 ± 5.46 and 81.88 ± 4.19 in the osteotomy guide robot group. The average values improved significantly compared with pre-operatively, but there was no statistical difference between the two robots. At 6 weeks post-operatively, the FJS was 63.6 ± 4.77 and 64.38 ± 4.60 in the guide plate-based robot and osteotomy guide robot groups, respectively, and there was no statistical difference between the two robots. At 24 months after surgery, the FJS of the two groups was 79.96 ± 5.22 and 80.98 ± 5.04, respectively, exhibiting significant improvement compared with that at 6 weeks, but there was still no statistical difference between the two groups (Table 4).

**Table 4:** Knee joint function in patients undergoing two robotic-arm-assisted TKA

Time	Outcome	Guide plate-based robot group	Osteotomy guide robot group	*p*-value
Before surgery	HSS	42.88 ± 2.91	43.46 ± 3.43	0.364
	SF-12	44.22 ± 4.27	43.12 ± 3.89	0.182
6 Weeks post-operative	HSS	65.1 ± 4.54	63.2 ± 4.82	0.045
	SF-12	62.08 ± 4.80	62.94 ± 4.82	0.374
	FJS	63.6 ± 4.77	64.38 ± 4.60	0.407
24 Months post-operative	HSS	78.96 ± 5.35	80.56 ± 5.46	0.142
	SF-12	80.54 ± 4.67	81.88 ± 4.19	0.134
	FJS	79.96 ± 5.22	80.98 ± 5.04	0.323

## Discussion

Surgery time is related to surgeon experience and the incidence of post-operative infectious complications [14]. Robot-assisted surgeries generally take longer than traditional manual surgeries [15]. In terms of robot use, after the learning curve reaches an inflection point, the initially prolonged surgery time shortens. All new technologies require sufficient learning. For example, the learning curve range of RATKA is 7–80 cases [16, 17]. Studies in the United Kingdom and Belgium have reported the learning curve of the MAKO robot. The inflection points where the surgery time levels off were at case 7. We found that with the osteotomy guide robot, the average operating time of surgeon 1 was 118.52 ± 15.95 min, which is similar to current reports [18]. However, the average operating time with the guide plate-based robot was 98.16 ± 9.68 min, which is much shorter than that of the osteotomy guide robot and similar to the currently reported manual operating time [1]. We also analysed the times for bone registration, the osteotomy, and the installation of the reference frame during the operation. We found that the guide plate-based robot group has a short osteotomy time. This may be related to the osteotomy guide plate module used with the guide plate-based robot; this module does not change the surgeon’s osteotomy habits. An osteotomy guide plate module will reduce the surgeon’s learning cost and shorten the osteotomy time [19, 20]. In this study, the learning curve of surgeon 1 for the osteotomy step with the guide plate-based robot reached an inflection point at case 5 versus case 9 with the osteotomy guide robot. For the surgeon 2, the total operating time with the two robots was the same. However, the osteotomy time of the first 10 cases shows that the average time is shorter for the guide plate-based robot group than the osteotomy guide robot group, indicating that the guide plate-based robot is easier to use and conforms to traditional surgical habits. The learning curves of the osteotomy time of the two robots of surgeon 2 reached inflection points at case 13. Although the timeframes were different, the inflection points on the learning curves occurred at the same point. Subsequently, both total operative time and osteotomy time demonstrated sustained declines. The average time was shorter for the last 10 cases than for the first 10 cases.

In this study, we used three scoring systems to evaluate patient satisfaction. The SF-12 score is widely used to evaluate overall health status and quality of life [21]. The HSS score is used to evaluate the clinical results of knee arthroplasty and has six main components: pain, function, range of motion, muscle strength, knee flexion deformity, and stability [22]. The FJS scoring system measures whether patients forget they have artificial joints after knee arthroplasty and is often used to measure the effect of joint arthroplasty [23]. We found that the patients who underwent robot-assisted TKA had a better HSS score, SF-12, and FJS than before surgery, although there was no significant difference between the osteotomy guide robot and guide plate-based robot groups. There may be two reasons for this. First, many factors affect knee joint performance and function, such as soft tissue balance, muscle relaxation, and patient complications. Second, both robots enable accurate osteotomies, and both operate in perfect accord with the surgeon’s decisions, so the effects are the same. The difference may also be due to the small sample size; therefore, long-term follow-up or a larger sample is needed to assess the improvement in clinical efficacy. Importantly, neither robot increased the risks of TKA.

Our study has limitations. Uncontrolled variability among suturing surgeons may have confounded total operative time measurements. Therefore, only the operating surgeon’s osteotomy duration, rather than total surgical time, was analysed in calculating the learning curve. And, steps such as array placement were performed by assistants, not the lead surgeons. The time variability during intra-operative adjustment of surgical planning was unstable, as the majority of the work was completed pre-operatively. Therefore, these steps lack standardized protocols, and learning curve analysis could not be conducted for them. Third, due to the limited utilization of ROSA robots in our institution, we selected a robot produced by a Chinese company for the guide plate-based robotic system, rather than the internationally established ROSA system. Fourth, as the TINAVI robotic system does not have its own dedicated prosthesis, we selected the widely used Depuy Attune prosthesis. Furthermore, since the Depuy VELYS robotic system is currently unavailable in China, a direct comparison with it was not feasible. Fifth, we were unable to prospectively collect the discrepancies between the pre-operative osteotomy thickness planning and the intra-operative real-time osteotomy block thickness for the MAKO robotic system, thereby preventing a comparative analysis with the TINAVI robotic system.

## Conclusions

Our study demonstrated that there was no significant difference in the learning curves for osteotomy procedures between the two robotic systems, despite differences in the time required. Furthermore, the cutting guide-based robot and the osteotomy-guide robot provided comparable levels of post-operative patient satisfaction.

## Data Availability

The datasets used and/or analyzed during the current study are available from the corresponding author on reasonable request.
